# Theory of Mind, Personal Epistemology, and Science Learning: Exploring Common Conceptual Components

**DOI:** 10.3389/fpsyg.2020.01140

**Published:** 2020-06-12

**Authors:** Natassa Kyriakopoulou, Stella Vosniadou

**Affiliations:** ^1^Department of Early Childhood Education, National and Kapodistrian University of Athens, Athens, Greece; ^2^College of Education, Psychology and Social Work, Flinders University, Adelaide, SA, Australia

**Keywords:** theory of mind, personal epistemology, science learning, conceptual change, observational astronomy

## Abstract

We investigated the hypothesis that theory of mind (ToM) and epistemological understanding promote the aspect of science learning that concerns the ability to understand that there can be more than one representation of the same phenomenon in the physical world. Sixty-three students ranging in age from 10 to 12 years were administered two false-belief ToM tasks, an epistemological understanding task that investigated beliefs about the nature of science and a science learning task. The science learning task required distinguishing and reflecting upon phenomenal and scientific depictions of phenomena in observational astronomy. A three-stage hierarchical multiple regression showed that ToM was a significant predictor of performance in the astronomy task, supporting the hypothesis of a common underlying conceptual component. The results also showed that performance in the personal epistemology–nature of science task was a significant predictor of performance in the astronomy task, even when ToM and age were taken into consideration. The results indicate that both ToM and epistemological understanding promote the ability to construct and reflect on phenomenal and scientific representations of the same situation in the physical world and have important implications for science education.

## Introduction

The learning of science is a complex task for many students, requiring the development of a host of interrelated thinking skills and conceptual changes (Carey, 1985; Chi, 2009; Kuhn, 2011; Vosniadou, 2019). The present research focuses on one aspect of this development, which has to do with the ability to construct and flexibly manipulate more than one representation of the same phenomenon in the world. For example, it has been shown that between the ages of 6 and 12, children recategorize their concept of the Earth from that of a physical object (a flat ground with the Sun and Moon in the sky above) to an astronomical object (a rotating sphere, a planet revolving around the Sun; Vosniadou and Brewer, 1992, 1994; Vosniadou et al., 2004; Vosniadou and Skopeliti, 2014). This recategorization suggests that the children had constructed a second, scientific representation of the Earth, which was distinct from their original, perception-based representation.

Recent research has shown that scientific representations do not supplant initial, phenomenal representations but coexist with them (Shtulman and Valcarcel, 2012; DeWolf and Vosniadou, 2015; Vosniadou et al., 2018). One of the important tasks of science learning and scientific thinking is to learn to navigate flexibly between such alternative representations (Pozo et al., 1999; Schwartz and Brown, 2013; Treagust et al., 2017). This is not an easy task. In a series of experiments, Kyriakopoulou and Vosniadou (Kyriakopoulou and Vosniadou, 2004, 2008; Vosniadou and Kyriakopoulou, 2006) presented elementary school children with phenomenal and scientific depictions of the same astronomical phenomena, such as the shape of the Earth, the structure of the solar system, and the day/night cycle, and asked them to select those that were closer to the way “things appear to be” and those that were closer to the way “things really are.” The results showed an increase in the selection of the depictions that represented scientific representations with development, indicating that the children had acquired some scientific knowledge. Having constructed a scientific representation does not necessarily imply, however, that science learning has been completed. Students still need to learn how to distinguish scientific from phenomenal representations and manipulate them appropriately depending on the context. The results of the above-mentioned studies showed that for the majority of the astronomical phenomena investigated, many children either mixed up the two kinds of representations, thinking that scientific depictions represented “the way things appear to be” and phenomenal depictions represented “the way things really are,” or selected only scientific depictions and said that they stood both for appearance and for reality. It was even more difficult for children to understand how the phenomenal depictions were related to the scientific ones and to explain why.

The development of scientific thinking skills, of which the ability to construct and manipulate multiple representations is one, has been attributed, in addition to content knowledge, to several other factors, such as information processing, executive function (working memory, shifting, and inhibition), and logical, spatial, and language abilities (e.g., Klahr, 2000; Bullock et al., 2009; Plummer, 2014; Koerber et al., 2015; Vosniadou et al., 2015; Plummer et al., 2016; Vosniadou et al., 2018). More recently, the development of scientific thinking skills has been linked to epistemological understanding (Carey et al., 1989; Carey and Smith, 1993; Kuhn, 2011), and even more recently to social cognition and theory of mind (ToM; Astington et al., 2002; Sodian et al., 2002; Osterhaus et al., 2017).

In the present research, we explore the links between children’s ability to construct and manipulate scientific representations in observational astronomy, their epistemological understanding, and social cognition and more specifically, the development of a ToM. We propose (1) that children’s ability to think about the differences between their beliefs and the beliefs of others in the social domain is a precursor of their ability to understand that the same event in the physical world can receive more than one interpretation and (2) that both of these abilities are related to the understanding of the constructive nature of knowledge—the understanding that our beliefs do not have an immediate relation to the world but are conjectures, hypotheses, that need to be verified and that they can be proven to be wrong. In the pages that follow, we describe the hypothesized links between ToM, personal epistemology (PE), and science learning in observational astronomy (SLOA) in greater detail.

### Links Between ToM and PE

Theory of mind research investigates the development of knowledge about one’s mental states such as beliefs, emotions, thoughts, and desires (Astington et al., 1988; Flavell, 2004; Apperly, 2010, 2012; Sodian and Kristen, 2016). Although children have some understanding of the basic concepts of intentionality (Meltzoff, 1995) and desire (Poulin-Dubois, 1999) from early on, they do not understand that people can have different beliefs about the same situation in the world until the age of about 4–5 years (Wimmer and Perner, 1983; Perner, 1988, 1991; Mitchell and Lacohee, 1991; Taylor et al., 1991). ToM knowledge continues to develop during the elementary school years as children come to understand that it is possible for other people to see something that they themselves cannot see and that the same object can receive different interpretations when viewed from different positions (Flavell et al., 1990; Flavell, 2000) or is seen by people with different prior knowledge (Mitchell and Lacohee, 1991; Perner, 1991; Taylor et al., 1991).

The possible links between ToM and epistemological understanding were first pointed out by researchers like Carpendale and Chandler (1996) and Kuhn et al. (2000), who argued that children’s achievements in ToM mark the first step toward an increasingly interpretive view of the nature of our mental world (see also Chandler and Carpendale, 1998, and Wellman et al., 2001). Smith et al. (2000) also argued that between the ages of 4 and 6, children develop the beginnings of a PE within the framework of their ToM and then continue to reconstruct their epistemological understandings as they encounter different knowledge claims in various domains. Based on the assumption that young children’s concepts are organized in intuitive theories that undergo conceptual change, Smith et al. situated children’s initial epistemology as a sub-theory within their initial ToM.

Empirical support for these theoretical arguments was provided in a study by Astington et al. (2002) who investigated relations between children’s performance in second-order ToM belief tasks and their epistemological understanding—namely, their ability to distinguish between the cause of a situation and a person’s reason for believing it. Seventy-four children between the ages of 5 and 7 were given two- second-order false-belief (FB) tasks and two evidence tasks. In the FB tasks, the children saw the enactment of a story about a protagonist who moves a letter from place A to a new location B while mistakenly believing her friend to be absent. The children were asked to predict where the protagonist thought the friend would look for the letter. In the evidence tasks, the children also saw the enactment of two stories and were asked about the cause of an event and the character’s evidence for it. For instance, in one story, a girl comes into the room and gets her feet wet without knowing that the floor was wet because a boy spilled water on it. The participants were asked why the girl’s feet were wet and whether the girl knew the reason why. The results showed that performance in the second-order FB tasks was correlated with performance in the evidence tasks and was a better predictor of them over general language and non-verbal abilities. The authors concluded that second-order FB understanding is significantly related to epistemological understanding as exhibited in children’s ability to make a distinction between two epistemologically distinct entities—i.e., the cause of an event and a person’s evidence for it.

### Links Between ToM, PE, and Science Learning

The links between epistemological understanding and science learning were made by researchers such as Hofer and Pintrich (1997) and Kuhn (2000, 2011) early on. A training study by Sodian et al. (2002) and a recent study by Osterhaus et al. (2017) provided empirical evidence for a relation between performance in a nature of science task and experimentation skills. Other related works suggest that there are relations between epistemological understanding and a wide range of scientific thinking skills and not only experimentation (Qian and Alvermann, 1995; Mason, 2000, 2003; Koerber et al., 2015; Sodian et al., 2016).

So far, ToM has been linked to the development of experimentation skills via its relation to epistemological understanding. As mentioned earlier, ToM can be helpful in the development of a PE because it leads to the recognition that empirical data and theory are distinct entities and therefore have a different epistemological status as sources of knowledge (Astington et al., 2002; Kuhn, 2011). The development of a PE is, in turn, a precursor of the development of experimentation skills. This hypothesis was examined by Osterhaus et al. (2017) who investigated relations between advanced theory of mind (AToM), nature of science understanding, experimentation skills, and general information processing (inhibition, intelligence, and language abilities) with 402 children aged 8 to 10 years. The results indicated that AToM was an important precursor of epistemological understanding, while, in turn, children’s epistemological understanding was a predictor of experimentation skills. Information processing abilities were also shown to be significantly related to experimentation skills.

The purpose of the present research was to further investigate links between advanced ToM, advanced epistemological understanding, and scientific thinking. The aspect of scientific thinking of interest in the present study was not experimentation skill but the ability to entertain dual representations of the same phenomenon in the world, and more specifically, phenomenal and scientific representations in observational astronomy. In agreement with prior research, we hypothesized that ToM would be a precursor of advanced epistemological understanding and that, in turn, PE might be a precursor of SLOA.

Furthermore, we hypothesized that there might be direct links between ToM and SLOA. Science learning requires that children can understand that the same phenomenon in the world can receive an interpretation different from that which is based on their phenomenal experience. For example, they must understand that although the Sun seems to hide behind mountains or clouds at night, the cause of the day/night cycle is to be found in the axis rotation of the Earth. They must also understand that the scientific and phenomenal interpretations do not function independently but are related to each other because they both refer to the same situation in the world, although in a different way. As mentioned earlier, the construction of a scientific representation does not mean that science learning has occurred. Only when children can both construct and reflect on different possible representations of the same phenomenon can we say that scientific learning has been achieved. As discussed earlier, the same ability underlies ToM development in the social domain. In other words, there seems to be a common cognitive/conceptual component that underlies both ToM and SLOA and that is related to the ability to construct and reflect on more than one representation of the same situation. Furthermore, the development of this ability in the social domain (ToM) appears to be a precursor of its development in the physical domain SLOA.

If there is a direct link between ToM and SLOA, what is then the role of epistemological understanding? A possible answer to this question is that epistemological understanding might create the ground—the foundation—that enables the transfer of knowledge from the social to the physical domains. ToM aids in the development of a PE because it helps children to become aware of the constructive nature of knowledge in general and the differences between theory and evidence (Sodian and Kristen, 2016). This metaconceptual understanding about the nature of knowledge, in turn, makes it more likely for children to notice that it can apply to domains other than ToM, namely, to people’s explanations of phenomena in the physical world.

**FIGURE 1:**
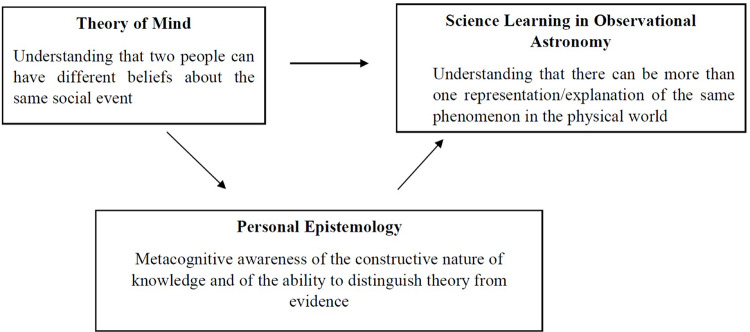
Hypothetical relations among ToM, PE, and SLOA.

To sum up, we hypothesized that the emerging awareness that people can have different beliefs about events in the social world in ToM is an important precursor in children’s ability to construct and reflect on different representations of the physical world SLOA. Furthermore, we hypothesized that both ToM and SLOA are conceptually linked to PE. ToM contributes to the emergence of PE as demonstrated by previous research. Developments in PE, in turn, facilitate the development of SLOA, allowing children to transfer their understanding of the possibility of alternative beliefs from social situations to the physical world. Figure 1 demonstrates the hypothetical relation between ToM ability, PE, and SLOA.

The hypotheses were tested by examining the relation between children’s performance in two ToM, one PE, and one SLOA tasks. Advanced ToM knowledge was investigated using two FB tasks in which the children had to set aside their knowledge of reality and to attribute an FB to an agent that lacked this knowledge. In the SLOA domain, children were administered a task in which they had to set aside their phenomenal perception of the physical world and adopt a scientific explanation. In both the ToM and SLOA tasks, the children were also asked to justify their responses and to reflect on what they knew and on how they knew it (Lombardi and Sinatra, 2018). The *Nature of Science Interview* (Carey et al., 1989; Smith et al., 2000; Smith and Wenk, 2006) was used to investigate epistemological understanding. We predicted that performance in all tasks will increase with age (Hypothesis 1). We also predicted that performance in the ToM tasks would be a significant predictor of performance in the SLOA task, even when PE and age were taken into account (Hypothesis 2). Finally, we predicted that performance in the PE task would be a significant predictor of performance in the SLOA task (Hypothesis 3).

## Materials and Methods

### Participants

The participants were 63 students (34 male) who attended grades 5 and 6 in a middle-class school in central Athens. They ranged in age from 10 to 12 years and 6 months (mean age: 10 years and 7 months). There were 27 children ranging in age from 10 years to 10 years and 11 months (mean = 10 years and 3 months), 26 children ranging in age from 11 years to 11 years and 11 months (mean = 11 years and 2 months), and 10 children ranging in age from 12 years to 12 years and 6 months (mean = 12 years and 1 month).

### Procedure

The students were interviewed individually at their school, in a quiet room, by one of the experimenters. The measures were administered in two sessions. The two ToM tasks and the PE task were administered first. The SLOA task was administered in the second session. Each session lasted approximately 30 to 40 min. All interviews were audio-recorded and were later transcribed for scoring.

### Materials

#### ToM Tasks

The materials consisted of the second-order FB task “Ice Cream Story” (Perner and Wimmer, 1985) and the third-order FB task “Double Bluff” by the Strange Stories (Happé, 1994). The second-order FB task was the “Ice-Cream Story.” In this story, there are two friends, John (agent A) and Mary (agent B) who want to buy ice cream. John knows that the ice cream van has been moved from a park to a church. Mary is also informed about the location change, but John does not know that Mary knows about the new location. The crucial question is where John thinks Mary will go to buy ice cream—i.e., to the park or to the church. In order to succeed in this task, the students must understand that John can have an FB about Mary’s belief. In other words, that it is possible to have beliefs about other people’s beliefs, and these beliefs may be false. As Perner and Wimmer (1985) discuss, there is a conflict between Mary’s propositional attitude (Mary knows the van is in the church) and Mary’s propositional attitude as believed by John (Mary thinks the van is in the park). There is also a conflict between what John believes about Mary’s belief and about what the child knows. In this case, the child must set aside his/her own knowledge about Mary’s current belief and must interpret and evaluate John’s model about the ice cream van’s current location.

The students were asked three questions. First, a comprehension question—“Where is the Ice-Cream Van?”—was asked in order to ensure that the story was understood. Second, a question was asked about John’s belief—“Where does John think Mary has gone?”—in order to determine whether they understood that John would believe that Mary would go to the park and not to the church. Third, the children were asked the question “Why does John believe that?” to justify why they thought that John had an FB.

Third-order belief tasks investigate more advanced forms of ToM that involve feelings, motives, and the use of more complex linguistic forms such as indirect speech, irony, and white lie (Happé, 1994). In the third-order FB task, students must understand that “the intended meaning of a message is different from the literal meaning of the utterance” (Miller, 2012). The “Double Bluff” story given to the students described a situation where a soldier is interrogated by the enemy about the location of his army’s tanks. In the process of the interrogation, the soldier reveals the true location of the tanks with the intention of deceiving the enemy. In other words, the soldier thinks that the enemy will not believe that he will reveal the tanks’ true location and will think that what he is telling them is not the true location of the tanks. In order to succeed in this task, students must understand that the soldier wants to deceive the enemy. In other words, the child must recognize that the soldier’s utterance is intended to be interpreted non-literally. As in the previous task, the participants were also asked three questions. First a comprehension question (“Is what the prisoner said true?”), second an FB question (“Where will the enemy look for the soldier’s army tanks?”), and third a justification question (“Why did the prisoner say what he said?”).

#### PE Task

The *Nature of Science Interview* (Carey et al., 1989; Smith et al., 2000; Smith and Wenk, 2006) was used to test students’ PE. This instrument examines the extent to which students have developed an understanding of the constructive nature of science. In a structured interview, the participants were asked to respond to four clusters of questions. Cluster 1 asked the students about the general aims of science and the types of questions scientists ask. Cluster 2 was about the nature and purpose of experiments and experimental procedures. Cluster 3 was about the nature of hypothesis formation and testing, and Cluster 4 was about the nature and the process of theory change. The exact questions that are used in the interview can be found in Table 1.

**TABLE 1:** The Nature of Science Interview (Carey et al., 1989; Smith et al., 2000; Smith and Wenk, 2006).

THE NATURE OF SCIENCE INTERVIEW
**1. General Aims of Science—Types of Questions**
1.1 Our friend John has some questions. He read some things about what science is about and what scientists do and wants our help to understand what he read. He also wants to hear what you think about science and scientists. What do you think the word “scientist” means? Can you give him an example?
1.2 What sorts of things do scientists do? How do they reach their goals?
1.3 Do scientists ask questions? Can you give me a specific example of a question that a scientist would ask?
1.4 What would scientists do to answer their question?
**2. Nature and Purpose of Experiments**
2.1 What is an experiment?
2.2 Do scientists do experiments? Why do scientists do them? In general, how do scientists decide what experiment to do?
**3. Nature of Hypotheses**
3.1 Many times, we make hypotheses about various things. Have you ever heard the word hypothesis? What do you think is a hypothesis a scientist does? Do you think a hypothesis is the same as a guess or do you think that there is a difference? What is the difference?
3.2 Can you give a specific example of a scientific hypothesis?
**4. Nature of Theories—Process of Theory Change**
4.1 Do scientists have ideas/theories about the world?
4.2 What is a scientific theory? Can you give a specific example of a scientific theory?
4.3 Do you think a scientist’s ideas influence the way he tries to find answers to his questions?
4.4 Say two scientists believe different things about our world. How can we decide which one is right?
4.5 Do scientists ever change their hypotheses or theories? When would they do that and why? Can scientists make mistakes or be wrong? How? Do scientists always achieve their goals? Why?


#### SLOA Task

The ability to reason about conflicting conceptual models of the physical world was measured using the SLOA task. The SLOA is a computer-based task that investigates students’ knowledge of the scientific representations in observational astronomy and their ability to distinguish them from perceptually based representations. It also investigates the ability to reflect on the discrepancies between these two representations. The task consists of two pictures of each of six astronomical phenomena (*Earth Shape*, *Gravity*, *Relative Size of Sun and Moon*, *Relative Size of Sun and Earth*, *Day/Night Cycle*, and *Solar System*), shown in Table 2. One picture depicted phenomenal representations and the other scientific representations. The pictures were based on previous research in which children were asked to create their own representations of the same astronomical phenomena (Vosniadou and Brewer, 1992, 1994; Vosniadou et al., 1996). The task was validated in an earlier study with sixth-grade children (Kyriakopoulou and Vosniadou, 2008) in which the children were asked to explain what astronomical phenomena these pictures represented.

**TABLE 2:** Science Learning in Observational Astronomy Task.

	SLOA task		
Astronomical phenomenon	*Question 1*: Look at the two pictures. What does the first picture show? What does the second picture show?		*Question 3*: Justification question
	***Question 2*: What are the differences between these two pictures?**		
Earth’s shape	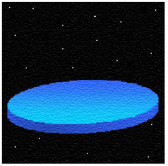	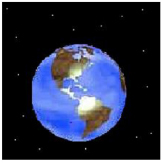	How do you explain that the Earth seems flat, when at the same time we accept that it is a sphere?
Where people live on the earth (Gravity)	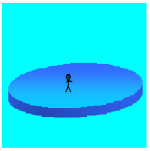	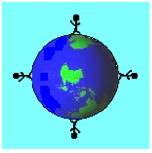	Since people can stay everywhere on Earth, can you tell me where this ball would fall if someone was here at the bottom of the Earth and here at the top of the Earth? (the experimenter shows where) Why is this happening?
Sun–Moon relative size	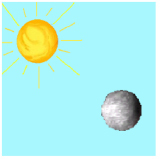	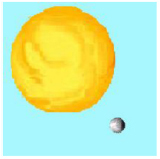	Why do the Sun and the Moon look like they have the same size, when in fact the Sun is bigger than the Moon?
Sun–Earth relative size	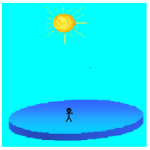	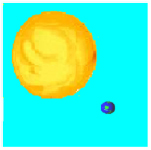	Why does the Sun seem smaller to us, when in fact it is much larger than the Earth?
Solar system	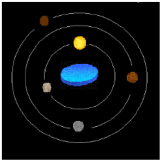	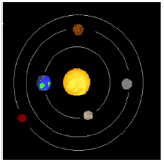	Why do we not understand the Earth’s movement around the Sun? Why do you say that the Earth moves when we do not feel its movement?
Day–Night cycle	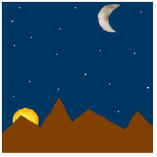	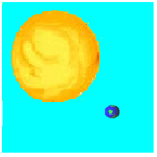	Why do we see the Sun rising from the East and setting from the west, when we know that it is the Earth that moves and not the Sun?

The students were shown the two pictures of each phenomenon in a random order. They were told that pictures were about the Earth, Sun, and Moon and the specific astronomical phenomenon investigated (e.g., the shape of the Earth, where people live on the Earth, the relative size of the Earth, and the Moon, etc.). They were then asked the following questions: (1) “Look at the two pictures. What does the first picture show? What does the second picture show? These questions tested children’s understanding of the referential nature of these pictures. They also revealed if they had been exposed to a scientific explanation of them.” (2) What are the differences between these two pictures? This question tested whether the children were able to understand that the two pictures referred to the same phenomenon in the world. If the students did not refer to the distinction between appearance and reality at the time of the second question, the experimenter prompted them to select which picture was closer to the way things appear to be and which picture showed the way things are in reality, clarifying that they could choose the same picture for both questions. Question 3—the justification question—was asked only if the students referred to the appearance–reality distinction in the second question. The justification questions for each phenomenon are shown in Table 2.

### Scoring

#### ToM Tasks

Responses to ToM tasks (Perner and Wimmer, 1985; Happé, 1994) were placed in five categories based on the recognition of FB and the type of justification provided in both tasks. The response categories were the following: no recognition of FB (score 1); recognition of second-order FB only (score 1.5); recognition of second-order FB and correct justification (score 2); recognition of both second- and third-order FBs and correct justification for the second-order belief (score 2.5); and recognition of second- and third-order FBs and correct justification for both (score 3). Two independent coders placed the students in one of the above-mentioned categories. Agreement rate was calculated to be 97% and was statistically significant based on Kendall’s tau correlation analysis (*τ* = 0.965, *N* = 63; *p* < 0.001). All disagreements were resolved through discussion. Two students recognized both the second- and third-order FBs but provided correct justification only for the third-order FB task, while three students recognized and correctly justified only the third-order FB. These students were placed in the more advanced category 5 because they succeeded in the most advanced task. The reliability of children’s responses in the second-order FB task and third-order FB task was not very high (Cronbach’s α = 0.43). This was probably due to the small number of items (two tasks—second and third order—and two responses per task, correct vs. incorrect recognition, and justification or not). Prior studies in which these tasks were used have shown that they were valid assessments of children’s ToM ability.

#### PE Task

The *Nature of Science Interview* task (Carey et al., 1989; Smith et al., 2000; Smith and Wenk, 2006) was scored based on the system developed by Carey and her colleagues (Carey et al., 1989; Carey and Smith, 1993; Smith et al., 2000; Smith and Wenk, 2006), consisting of four question clusters, each of which had five epistemic levels. Table 3 shows the type of student responses for each question cluster by epistemic level.

**TABLE 3:** Type of student responses in each question cluster in the Nature of Science Interview by epistemic level.

Cluster of questions	Epistemic level				
	Level 1	Level 1.5	Level 2	Level 2.5	Level 3
Cluster 1: Q1.1–1.4 General aims of science and type of scientists’ questions	Scientists simply find or discover new information and ask procedural and journalistic questions	Scientists try to find out how something works (unclear if they refer to a procedure or a mechanism)	Scientists try to find underlying mechanisms, ask questions about theoretical entities and reflective questions about their ideas	Scientists formulate questions to find out how something works	Discuss how multiple levels of questions interact
Cluster 2: Q2.1–2.2 Nature and purpose of experiments and experimental procedures	Experiment is similar to producing a desirable outcome	Experiment involves measuring variables/scientists do experiments to find out how something works	Scientists test their ideas by an experiment	Experiments are a way to test competing hypotheses	Experiments test causal ideas
Cluster 3: Q3.1–3.2 Nature of hypothesis formation and theory testing	No differentiation between hypotheses and experiments	Hypotheses are similar to guesses	Scientists understand and evaluate their own ideas through experimentation	Hypotheses are defined as explanatory ideas	Hypothesis testing provides evidence for/against a theory
Cluster 4: Q4.1–4.7 Nature and process of theory change	Scientists may abandon or change an idea based on a single experiment or observation	Hypotheses can change, but theories do not, and change occurs by doing experiments	Scientists develop new ideas	The ideas scientists investigate are more complex, and it takes work to understand them	Ideas are embedded in theoretical frameworks that constrain the generation of new hypotheses

Each epistemic level was scored as 1, 1.5, 2, 2.5, and 3, respectively. Two coders independently scored all of students’ responses. Agreement rate was calculated to be 98% for Cluster 1 (τ = 0.976, *N* = 63; *p* < 0.001), 98% for Cluster 2 (τ = 0.985, *N* = 63; *p* < 0.001), 94% for Cluster 3 (τ = 0.940, *N* = 63; *p* < 0.001), and 92% for Cluster 4 (τ = 0.921, *N* = 63; *p* < 0.001). All disagreements were resolved through discussion. Reliability for children’s responses for all the 13 questions was Cronbach’s α = 0.70.

Each student was given one score for each cluster, based on the highest epistemic level achieved in the cluster questions. A final average epistemic level score for each student was also computed based on the students’ level scores in the four clusters. The final epistemic level scores represented the following competencies: epistemic level 1 responses (*knowledge unproblematic epistemology*) agree with the belief in true and certain knowledge. Students refer to scientists’ ideas, experiments, and results in an undifferentiated mode, and goals are the activities and products of science. Epistemic level 1.5 responses are more elaborated concepts of level 1. Students become increasingly aware that scientists have ideas but do not yet understand that these ideas are tested through experimentation. Epistemic level 2 responses show sensitivity to explanation and hypothesis testing. Students begin to differentiate between ideas, experiments, and results. Epistemic level 2.5 responses are more complex and sophisticated expressions of level 2. The students at this level show a more complex understanding of the testing process with reference to multiple pieces of evidence and begin to understand that the development of ideas is not just a process of simply adding new ideas to preexisting ones. Epistemic level 3 responses (*knowledge problematic epistemology*) reveal an understanding of the uncertain and relative nature of knowledge (for a detailed description, see also Carey and Smith, 1993; Smith et al., 2000).

#### SLOA Task

A fourth-step process was followed to score the students’ responses with the SLOA task. First, for each astronomical phenomenon, each student selected one picture for appearance and one for reality. Based on their choices, the students were placed in one of the following four categories: (1) no distinction between appearance and reality—when the picture chosen was the phenomenal one for both reality and appearance; (2) appearance–reality reversed—when the picture chosen was the phenomenal one for reality and the scientific one for appearance; (3) scientific responses only—when the picture chosen was the scientific one for both reality and appearance; and (4) distinction—when the picture chosen was the scientific one for reality and the phenomenal one for appearance. Two coders independently scored all responses. There were no disagreements between the two independent coders (τ = 1, *n* = 63; *p* < 0.001).

Second, for each astronomical phenomenon, the students were asked to justify their choices. Depending on the type of the justification provided, their responses were placed in the following three categories: (1) no justification, if they could not make the appearance–reality distinction; (2) initial, if they were consistent with phenomenal experience or if they revealed any kind of phenomenal misunderstanding; and (3) scientific, if they could justify the differences between appearance and reality in scientific terms. Kendall’s tau correlation analysis showed that the agreement between the coders was statistically significant (τ = 0.977, *n* = 63; *p* < 0.001). All disagreements were resolved through discussion.

Third, based on the pictures selected and on their justifications, the children were placed in one of the five overall SLOA categories and were given the overall scores 1, 1.5, 2, 2.5, and 3, respectively, for each astronomical phenomenon, as shown in Table 4. In this step, there was total agreement between the coders in how they applied the five-level coding system (τ = 1, *n* = 63; *p* < 0.001).

**TABLE 4:** Presentation of the scoring process for each astronomical phenomenon in the SLOA Task.

First step			Second step	Third step	
	
Appearance–Reality distinction			Type of justification	Final total categories	
	Pictures chosen				
Response categories	Appearance	Reality	Response categories	Response categories	Overall score
No distinction	Phenomenal	Phenomenal	No justification	No distinction/no justification	1
A–R reversed	Scientific	Phenomenal	No justification	A–R reversed/no justification	1.5
Scientific responses	Scientific	Scientific	No justification	Scientific responses/no justification	2
Distinction	Phenomenal	Scientific	Initial justification	Distinction/initial justification	2.5
			Scientific justification	Distinction/scientific justification	3

Fourth, a final score for each student was also calculated based on the mean score in the combined six astronomical phenomena, and this final score was used in the statistical analyses. The reliability of children’s final SLOA responses for the six astronomical phenomena was α = 0.76.

## Results

### Descriptive Statistics

Table 5 shows the frequency and percentage of students assigned in the final response categories in the ToM task as a function of age.

**TABLE 5:** Frequency and percentage of students in the five categories of the two ToM tasks combined as a function of age (Perner and Wimmer, 1985; Happé, 1994; *N* = 63).

Response categories for ToM tasks		Age variation			
		10 *N* = 27	11 *N* = 26	12 *N* = 10	*Total N* = *63*
1	No recognition of false belief	5 (19%)	5 (19%)	1 (10%)	*11 (18%)*
1.5	Recognition of second-order false belief only	7 (26%)	5 (19%)	–	*12 (19%)*
2	Recognition of second-order false belief and correct justification	9 (33%)	3 (12%)	4 (40%)	*16 (25%)*
2.5	Recognition of both second-order and third-order false belief and correct justification for the second-order belief	–	1 (4%)	1 (10%)	*2 (3%)*
3	Recognition of second- and third-order false belief and correct justification for both	6 (22%)	12 (46%)	4 (40%)	*22 (35%)*

Table 6 shows the frequency and percentage of students assigned to each epistemic level in the four question clusters. As can be seen, the majority of the students were categorized at epistemic level 1. When looking at the performance of the students as a function of age, we can see (Table 7) an increase in epistemic level as predicted, particularly for the older students in the sample.

**TABLE 6:** Frequency and percentage of students in the four clusters of questions as a function of epistemic level at the *Nature of Science Interview* (Carey et al., 1989; Smith et al., 2000; Smith and Wenk, 2006; *N* = 63).

		Clusters of questions			
Epistemic level in the nature of science interview		Cluster 1: Q1.1–1.4 General aims of science and type of scientists’ questions	Cluster 2: Q2.1–2.2 Nature and purpose of experiments and experimental procedures	Cluster 3: Q3.1–3.2 Nature of hypothesis formation and theory testing	Cluster 4: Q4.1–4.7 Nature and process of theory change
1	Level 1: Knowledge unproblematic epistemology	52 (83%)	52 (83%)	52 (83%)	42 (67%)
1.5	Elaborated Level 1	4 (6%)	2 (3%)	10 (16%)	20 (32%)
2	Level 2: Transitional ideas: Introduction of explanation and hypothesis testing	7 (11%)	9 (14%)	1 (2%)	1 (2%)
2.5	Elaborated Level 2	–	–	–	–
3	Level 3: Knowledge problematic epistemology	–	–	–	–

**TABLE 7:** Frequency of students in each epistemic level based on their total responses in the Nature of Science Interview as a function of age (*N* = 63).

Epistemic level in the nature of science interview		Age variation			
		10 *N* = 27	11 *N* = 26	12 *N* = 10	*Total N* = *63*
1	Level 1: Knowledge unproblematic epistemology	26 (96%)	21 (81%)	5 (50%)	*52 (82%)*
1.5	Elaborated Level 1	1 (4%)	3 (11%)	2 (20%)	*6 (10%)*
2	Level 2: Transitional ideas: Introduction of explanation and hypothesis testing	–	2 (8%)	3 (30%)	*5 (8%)*
2.5	Elaborated Level 2	–	–	–	–
3	Level 3: Knowledge problematic epistemology	–	–	–	–

Table 8 shows the frequency and percentage of students placed in each of the five response categories as a function of age. As predicted, performance increased with age, particularly in the number of children who could provide justifications of the distinction between phenomenal and scientific depictions.

**TABLE 8:** Frequencies and percentage of students in the five categories based on their total responses in the SLOA Task as a function of age (*N* = 63).

Response categories for observational astronomy task		Age variation			
		10 *N* = 27	11 *N* = 26	12 *N* = 10	*Total N* = *63*
1	No Appearance–Reality distinction/No justification	4 (15%)	–	–	*4 (6%)*
1.5	Appearance–Reality reversed/No justification	2 (7%)	2 (8%)	–	*4 (6%)*
2	Scientific responses only/No justification	7 (26%)	5 (19%)	1 (10%)	*13 (21%)*
2.5	Distinction/Initial Justification	14 (52%)	14 (54%)	5 (50%)	*33 (52%)*
3	Distinction/Scientific Justification	–	5 (19%)	4 (40%)	*9 (14%)*
*Total*		*27*	*26*	*10*	*63*

### Correlations

Children’s final scores in the three tasks were considered to represent meaningful and equally spaced intervals indicating progressively higher levels of response. Pearson correlations showed significant results between age and performance in all the tasks: ToM tasks (*r* = 255, *p* < 0.05), PE (*r* = 468, *p* < 0.01), and SLOA task (*r* = 440, *p* < 0.01). Table 9 shows Pearson product–moment correlations between age (range of age: 10 years to 12 years and 6 months) and scores on ToM tasks (*M* = 2.09, *SD* = 0.76), Nature of Science Interview (PE; *M* = 1.14, *SD* = 0.20), and SLOA task (SL; *M* = 2.23, *SD* = 0.39). As hypothesized, performance in ToM, PE, and SLOA correlated significantly with each other, and all were correlated with age. Because of the skewness in the PE variable, the correlations were also conducted using the logarithmic transformation of the PE. The results did not change.

**TABLE 9:** Pearson correlations of age, ToM, PE, and SLOA (*N* = 63).

	Age	ToM	PE	SLOA
Age	–			
ToM	0.255*	–		
PE	0.468**	0.431**	–	
SLOA	0.440**	0.474**	0.531**	–

*p ≤ 0.05. **p ≤ 0.01.

### Regressions

Two hierarchical multiple regression analyses were conducted in order to examine the following hypotheses: (1) that both ToM and PE predict students’ performance in the SLOA task and (2) that both independent variables (ToM and PE) will continue to be good predictors for the dependent variable (SLOA) even when age was taken into consideration.

A two-step hierarchical regression analysis examined the first hypothesis. In this analysis, performance in the SLOA task was the dependent variable. We first introduced into the equation performance in the ToM tasks as a predictor. At the second step, performance in PE task was added. The results showed that at Step 1, ToM ability contributed significantly to the regression model [*F*(1, 62) = 17.707, *p* ≤ 0.001], and accounted for 23% of the variation in the SLOA task. The introduction of the PE variable explained an additional 13% of the variation in performance in the SLOA task [*F*(2, 62) = 16.558, *p* ≤ 0.001]. In the final equation, PE made a greater contribution (β = 0.401, *p* ≤ 0.001) than ToM (β = 0.302, *p* ≤ 0.001) (see Table 10).

**TABLE 10:** Summary of hierarchical regression analysis predicting performance on the SLOA Task from ToM and PE.

Step/variable added	*B*	SEB	β	*R*	*R*^2^	Δ*R*	Δ*F*
Step 1: ToM	0.249	0.059	0.474**	0.474	0.225		
Step 2				0.596	0.356	0.131	12.167**
ToM	0.159	0.060	0.302*				
PE	0.799	0.229	0.401*				

*p ≤ 0.01. **p ≤ 0.001.

A three-step hierarchical multiple regression analysis was conducted in order to examine the second hypothesis. In this analysis, performance on the SLOA task was the dependent variable. We first introduced into the equation the variable *age* as predictor. At the second step, performance in the ToM tasks was added. At the third step, performance in PE task was added in addition to age and ToM. A significant regression equation was found in all three steps (see Table 11). In Step 1, age was found to contribute significantly to the prediction of performance on the SLOA task [*F*(1, 62) = 14.632, *p* ≤ 0.001), explaining 19% of the variance, In Step 2, the introduction of ToM ability explained an additional 14% of the variance [*F*(2, 62) = 15.020, *p* ≤ 0.001]. The inclusion of PE in Step 3 also produced a significant regression equation [*F*(3, 62) = 12.851, *p* ≤ 0.001], explaining an additional 6% of the variance. As we see in Table 11, in the final equation, PE made a greater contribution (β = 0.301, *p* ≤ 0.01) than ToM (β = 0.287, *p* ≤ 0.01) whereas age made the least contribution (β = 0.226, NS).

**TABLE 11:** Summary of hierarchical regression analysis predicting performance on the SLOA Task from Age, ToM and PE.

Step/variable added	*B*	SEB	β	*R*	*R*^2^	Δ*R*	Δ*F*
Step 1: Age	0.252	0.066	0.440***	0.440	0.193		
Step 2				0.578	0.334	0.140	12.621**
Age	0.195	0.062	0.341**				
ToM	0.203	0.057	0.387**				
Step 3				0.629	0.395	0.062	6.007*
Age	0.129	0.066	0.226				
ToM	0.151	0.059	0.287**				
PE	0.601	0.245	0.301**				

*p ≤ 0.05. **p ≤ 0.01. ***p ≤ 0.001.

Both regressions were repeated using the logarithmic value for the PE variable. The results did not change.

**FIGURE 2:**
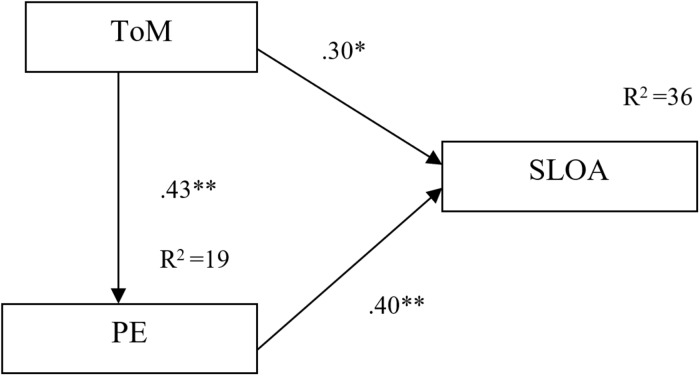
Path model showing a direct path from ToM to PE and from PE to SLOA, as well as a direct path from ToM to SLOA.

A path analysis using the Analysis of Moment Structure (AMOS 26) was used to test the hypothesized direct effects from ToM to SLOA and the mediating role of PE. The analysis resulted in a saturated, just-identified model (χ^2^ = 0, *df* = 0; GFI = 1, CFI = 1, and NFI = 1), indicating a perfect fit. All path coefficients were statistically significant, indicating positive direct effects from ToM on SLOA (β = 302, *p* ≤ 0.01), from PE on SLOA (β = 401, *p* ≤ 0.001), and from ToM on PE (β = 431, *p* ≤ 0.001) (see Figure 2). The indirect effect of ToM on SLOA through PE was also statistically significant (*p* ≤ 0.01, 95% Cl [0.074, 0.296]).

## Discussion

Previous research has examined relations between social cognition, epistemological understanding, and scientific thinking, focusing on experimentation. The focus of the present research was on a different aspect of scientific thinking that has to do with the ability to construct and reflect on more than one representation of the same phenomenon in the physical world. Many researchers have discussed the importance of the ability not only to construct but also to move accurately and flexibly among various levels of representations for science learning and conceptual change (Spada, 1994; Pozo et al., 1999; Kozma, 2003; Wiser and Smith, 2009). Recent research regarding the coexistence of phenomenal and scientific representations (Shtulman and Valcarcel, 2012) and the interference of phenomenal representations in scientific reasoning tasks with adults (Vosniadou et al., 2018) further underscores the importance of developing students’ ability to think with more than one representation. In the present study, we examined children’s ability to construct and reflect on phenomenal and scientific representations in observational astronomy, a domain of science whose development is well understood based on prior research.

Science learning in observational astronomy requires that children construct explanations of phenomena such as the shape of the Earth and the day/night cycle, which are different from those suggested by perceptual experience. It also requires that they understand how this scientific representation is related to their phenomenal experience. We argued that there are some similarities between this type of science learning and correct performance in second- and third-order ToM tasks. ToM tasks are usually based on the understanding that two people can have different beliefs about the same social event or that the same proposition may have two meanings, one literal and one non-literal, depending on the context (see also Moll and Tomasello, 2007; Tomasello and Moll, 2010). In other words, ToM and science learning seem to share a common underlying cognitive/conceptual component, having to do with the ability to construct and reflect upon two representations of the same situation. It was hypothesized that this ability will develop with age and that its development in the social domain would be a precursor of its development in science learning.

The results of the present research showed age-related increases in performance in both the ToM and the science learning tasks (Hypothesis 1). Regarding the ToM tasks, the findings revealed a significant correlation between ToM and age. The younger children (10-year-olds) were able to answer correctly the second-order belief task but found it difficult to justify their responses. Third-order FB understanding with correct justification increased in the 12- to 12.5-year-old group. These results are in accordance with existing research (Happé, 1994; Muris et al., 1999; White et al., 2009). Children’s developing ability to reflect on their reasoning (Miller, 2012) has been shown to be related to the ability to understand and imagine multiple perspectives and alternatives (Bosacki and Astington, 1999) and to epistemological thinking (Mason, 2002).

Regarding the astronomy task, the results also showed significant improvements with age. Sixty-six percent of the students distinguished the depiction that best represented “the way things appear to be” from “the way things really are” across the various astronomical phenomena (Categories 4 and 5), indicating that they knew the scientific explanation. However, the ability to verbally articulate the relation between phenomenal and scientific depictions increased, particularly in the 12- to 12.5-year age group. The results agree with previous research (Vosniadou and Kyriakopoulou, 2006).

Successful performance in the astronomy task undeniably requires domain-specific content knowledge as well as the development of complex perspective taking, spatial reasoning, information processing, and executive function skills. However, it also critically depends on the ability to understand that it is possible to entertain more than one representation of the same phenomenon in the physical world and reflect on these representations. The results of the regression confirmed that performance in the ToM tasks was a significant predictor of performance in the astronomy task. The present study is the first to show that ToM is a precursor of science learning. Previous research showed ToM to be a precursor of epistemological understanding only, but not of scientific thinking. This result supports the hypothesis that there is a common conceptual component between ToM and SLOA. Both tasks require the ability to construct and reflect upon dual representations. It appears that this ability develops first in the social domain. Understanding that people have different beliefs about the same social event facilitates the recognition that it is possible that the same phenomenon in the physical world might receive different interpretations.

Students’ ideas about the nature of science (Carey et al., 1989; Smith et al., 2000; Smith and Wenk, 2006) were found to be mostly in agreement with an initial level of PE (level 1), indicating an attachment to a single, true, and certain truth. These results are consistent with the findings of Carey et al. (1989); Honda (1994), and Smith et al. (2000) who used the same measure to investigate the epistemic beliefs of students attending the last grades of primary school and first grades of secondary school. In the present research, only a few of the older students (12–12.5 years old) gave responses that were categorized in level 2. It seems that for these few children, there is a small, although significant, shift from an entirely objective and certain view of knowledge to a more constructivist epistemological stance, where there is a need to think explicitly about their beliefs, examine them in a framework of alternatives, and provide the evidence to confirm/disconfirm them (Kuhn, 1993). The development of these dispositions to think about knowledge, the nature of science, and the process of knowing in a framework of conflicting views may serve as the foundation for the development of scientific thinking (Qian and Alvermann, 1995; Mason, 2000). Indeed, the results of the regression analysis confirmed the hypothesis regarding the relations between ToM, science learning, and epistemological understanding, by showing that performance in the nature of science task was a significant predictor of performance in the astronomy task, even when ToM and age were taken into consideration, explaining the largest percentage of the variance (Hypothesis 3).

A possible interpretation of the role of PE is that it acts as a mediator between ToM and SLOA, allowing the transfer of knowledge from the social to the physical domains. In other words, the recognition, first achieved in the ToM domain, that people can have different beliefs or that the same event may be interpreted in different ways becomes the foundation that allows children to form an awareness of the constructive nature knowledge in general (Astington et al., 2002; Eisbach, 2004; Sodian and Kristen, 2016; Osterhaus et al., 2017). It can also serve as the foundation for the recognition that evidence and theory are distinct entities, an understanding that is central to scientific learning (Kuhn et al., 2000; Iordanou, 2016). The development of a PE, in turn, further enables and facilitates the transfer of ToM understanding in the domain of science. Although a preliminary investigation of the possible mediating role of PE between ToM and SLOA was confirmed, this relation needs to be investigated in greater detail in future research with a larger number of participants and taking into effect especially the role of age and of executive function skills.

The awareness that different people can have different interpretations of the same social event and the ability to verbally articulate it and generalize it to other knowledge situations is not a trivial task. It constitutes a major cognitive development and requires considerable conceptual changes, similar to that described by Karmiloff-Smith (1992) in the area of language development. Karmiloff-Smith investigated how children treat the fact that the same word may have more than one meaning, i.e., that it can refer to two different situations in the world. Although in our case children face a reverse problem (where two different representations refer to the same situation in the world), nevertheless, in both cases, we have a problem that demands dealing with dual representations of some form. Karmiloff-Smith’s studies (1979) showed that 5- to 6-year-olds were not always able to use the words correctly and explain why. Only later, around the age of 6–7 years, were they able to do so, suggesting that they had achieved a consciously accessible and verbally stated metalinguistic knowledge. Karmiloff-Smith (1992) argued that this achievement was the product of a process of “representational re-description.” Through this process, implicit information becomes explicit knowledge, progressively available to other parts of the cognitive system and under self-evaluation. Then, it is feasible for the child to produce and use multiple representations at different levels of explicitness and detail.

In more recent work, Kuhn (2006) relates similar achievements to developments in executive control in adolescence that allow metacognitive reflection of one’s representations and flexible access to dual representations. According to Kuhn, the absence of this ability leads to a singular experience of “the way things are” as a framework for understanding the world.

The results of the present research indicate that level 2 epistemological understanding was only beginning to be achieved in the 12-year-old group. As the regression analysis showed, epistemological understanding was the most important predictor of the participants’ ability to reflect on conflicting astronomical depictions. The findings support the interpretation of the possible mediating role of PE and a conceptual link of ToM, science learning, and epistemological understanding. As children moved to a more constructivist epistemology of science, they succeeded more often in forming scientific representations and understanding their relation to their phenomenal experience.

### Limitations and Future Directions

The present research provides us with an initial understanding of the links between social cognition, epistemological understanding, and science learning in the area of observational astronomy but has several limitations. One limitation is the small sample size and the limited statistical analysis. The research needs to be replicated with a larger developmental sample that would allow the use of structural equation modeling to further test the presence of direct links between ToM and science learning, the hypothesized mediating role of epistemological knowledge, and the roles of age and executive function skills. In addition, other important factors such as spatial and perspective-taking abilities, language abilities, and prior knowledge should be considered in future research. It is very probable that particularly executive function skills, such as working memory, inhibition, and shifting, could account for part of the commonalities observed between ToM, PE, and SLOA. Future research needs also to further test the links between ToM and science learning in other domains of science, where there is reason to believe that common conceptual components are shared, not only in the domain of observational astronomy.

It is possible that the relations between social cognition, epistemological understanding, and science learning are bidirectional and not unidirectional. For example, it is possible that developments in students’ PE allow for a more advanced understanding of ToM, while the learning of science makes possible the development of more sophisticated epistemological understandings. This hypothesis needs to be tested in future research.

Last but not least, it is important to test the relations between ToM, epistemological understanding, and science learning using training and intervention experiments. If ToM and PE are precursors of science learning, we should expect that training in ToM, and/or ToM and PE at an early age improves children’s ability to construct and flexibly manipulate different representations of the same phenomenon in the physical world.

### Implications for Science Instruction

Many researchers have argued that science education should be oriented toward the development of students’ ability to construct multiple representations and be able to move flexibly among them (Spada, 1994; Pozo et al., 1999; Wiser and Amin, 2001; Treagust et al., 2017). According to Schwartz and Brown (2013), improvements in students’ ability to move across various levels of representation (e.g., phenomenal, molecular, and symbolic) could lead to greater scientific understanding. Children’s performance in the present observational astronomy task indicates that children, even when they have knowledge of the scientific representations, still find it difficult to navigate between them and the phenomenal ones and reflect upon them. Other research have also shown that students’ ability to integrate the use of multiple representations during science learning is limited. Students tend to focus on surface features and ignore underlying mechanisms and/or are unable to coordinate between different representations (Kozma, 2003; Seufert, 2003; Won et al., 2014).

It has been difficult to develop science instruction that can improve students’ ability to form flexible scientific representations and understand their relation to perceptual experience. The importance of information processing and executive function limitations, such as working memory, inhibition, shifting, and spatial reasoning, has often been emphasized. There is no doubt that these are very important. There have been few suggestions, however, as to how to strengthen children’s conceptual understanding. The present results suggest that instruction that focuses on ToM and PE might help children develop the conceptual understanding necessary to grasp the constructive nature of knowledge and the distinction between theory and evidence, paving the way for improved learning in science.

## Data Availability Statement

The datasets generated for this study are not publicly available. Requests to access the datasets should be directed to the corresponding author.

## Ethics Statement

The study was reviewed and approved by the Institute of Educational Policy of the Greek Ministry of Education and Religious Affairs. Written informed consent to participate in this study was provided by the participants’ legal guardian.

## Author Contributions

NK and SV contributed to the conception and design of the study. NK acquired the data and organized the database. NK and SV analyzed and interpreted the data and wrote sections of the manuscript. All authors contributed to manuscript revision and read and approved the submitted version.

## Conflict of Interest

The authors declare that the research was conducted in the absence of any commercial or financial relationships that could be construed as a potential conflict of interest.
